# Desensitization With Imlifidase for HLA-Incompatible Deceased Donor Kidney Transplantation: A Delphi International Expert Consensus

**DOI:** 10.3389/ti.2024.13886

**Published:** 2025-01-06

**Authors:** Lucrezia Furian, Uwe Heemann, Mats Bengtsson, Oriol Bestard, Isabelle Binet, Georg A. Böhmig, John Boletis, David Briggs, Frans H. J. Claas, Lionel Couzi, Emanuele Cozzi, Marta Crespo, Aiko P. J. De Vries, Fritz Diekmann, Magdalena Durlik, Denis Glotz, Ilkka Helantera, Annette Jackson, Stanley C. Jordan, Dirk Kuypers, Carmen Lefaucheur, Christophe Legendre, Tomas Lorant, Umberto Maggiore, Nizam Mamode, Smaragdi Marinaki, Annick Massart, Thomas Müller, Rainer Oberbauer, Lutz Renders, Dave Roelen, Jean-Luc Taupin, Ondřej Viklický, Angeliki Vittoraki, Annelies E. de Weerd, Maarten Naesens

**Affiliations:** ^1^ Kidney and Pancreas Transplantation Unit, Department of Surgery, Oncology and Gastroenterology DISCOG, University Hospital of Padova, Padova, Italy; ^2^ Abteilung für Nephrologie, Klinikum Rechts der Isar, Technische Universität München, Munich, Germany; ^3^ Department of Immunology, Genetics and Pathology, Uppsala University, Uppsala, Sweden; ^4^ Nephrology and Kidney Transplant Department, Vall d’Hebron University Hospital, Barcelona, Spain; ^5^ Clinic of Nephrology and Transplantation Medicine, Cantonal Hospital St Gallen, St. Gallen, Switzerland; ^6^ Division of Nephrology and Dialysis, Department of Medicine III, Medical University Vienna, Vienna, Austria; ^7^ Department of Nephrology and Renal Transplantation, National and Kapodistrian University of Athens, Laiko Hospital, Athens, Greece; ^8^ Histocompatibility and Immunogenetics Laboratory, Birmingham Centre, NHS Blood and Transplant, UK NHS Blood and Transplant, Birmingham, United Kingdom; ^9^ Eurotransplant Reference Laboratory, Department of Immunohaematology and Blood Transfusion, Leiden University Medical Center, Albinusdreef, Netherlands; ^10^ Department of Nephrology, Transplantation, Dialysis and Apheresis, Bordeaux University Hospital, Bordeaux, France; ^11^ Transplant Immunology Unit, Department of Cardiac, National Transplant Centre (CNT), Thoracic and Vascular Sciences Padua University Hospital - Ospedale Giustinianeo, Padova, Italy; ^12^ Department of Nephrology, Hospital del Mar, Nephropathies Research Group, Hospital del Mar Research Institute, Barcelona, Spain; ^13^ Division of Nephrology, Department of Medicine, and Leiden Transplant Center, Leiden University Medical Center, Leiden, Netherlands; ^14^ Department of Nephrology and Kidney Transplantation, Hospital Clínic Barcelona, Barcelona, Spain; ^15^ Klinika Transplantologii, Immunologii, Nefrologii i Chorób Wewnętrznych Warszawski Uniwersytet Medyczny ul, Warszawa, Poland; ^16^ Department of Nephrology and Renal Transplantation, Saint-Louis Hospital in Paris, Paris, France; ^17^ Transplantation and Liver Surgery, Helsinki University Hospital and University of Helsinki, Helsinki, Finland; ^18^ Department of Surgery, Duke University, Durham, NC, United States; ^19^ Nephrology and Transplant Immunology Medical Director Kidney Transplant Program Cedars-Sinai Medical Center, Pediatrics and Medicine David Geffen School of Medicine at UCLA, Los Angeles, CA, United States; ^20^ Department of Nephrology and Renal Transplantation, University Hospitals Leuven, Leuven, Belgium; ^21^ Department of Microbiology, Immunology and Transplantation, KU Leuven, Leuven, Belgium; ^22^ Nephrologist and Head of the Nephrology and Kidney Transplantation Department, Saint-Louis Hospital-APHP, Paris, France; ^23^ Nephrology at Université Paris Cité and Head of Nephrology and Transplantation Unit at Necker Hospital in Paris, Paris, France; ^24^ Uppsala University, Department of Surgical Sciences, Section of Transplant Surgery, Uppsala, Sweden; ^25^ Dipartimento di Medicina e Chirurgia, Università di Parma, UO Nefrologia - Trapianti Rene Pancreas, Programma Regionale Trapianti Emilia-Romagna, Azienda Ospedaliero-Universitaria di Parma, Parma, Italy; ^26^ Department of Transplantation, Transplant Surgery at Guy’s and Great Ormond Street Hospitals, London, United Kingdom; ^27^ National and Kapodistrian University of Athens, Clinic of Nephrology and Transplantation, “Laiko” General Hospital, Athens, Greece; ^28^ Department of Nephrology, UZ Antwerpen, Antwerpen, Belgium; ^29^ Clinic for Nephrology, Renal Transplant Program, Transplant Institute, University Hospital Zurich, Zurich, Switzerland; ^30^ Medical University of Vienna, Vienna, Austria; ^31^ Department of Nephrology of Technische Universität München, München (TUM), Munich, Germany; ^32^ Department of Immunology, Leiden University Medical Center, Leiden, Netherlands; ^33^ Laboratory of Immunology and Histocompatibility, Hôpital Saint-Louis, APHP Paris, Paris, France; ^34^ Department of Nephrology, Institute for Clinical and Experimental Medicine, Prague, Czechia; ^35^ Immunology Department and National Tissue Typing Center, “G.Gennimatas” Hospital, Athens, Greece; ^36^ Erasmus MC Transplant Institute, Department of Internal Medicine, University Medical Center, Rotterdam, Netherlands

**Keywords:** desensitization, HLA incompatible, HLAi, kidney transplantation, imlifidase

## Abstract

Highly sensitized (HS) patients in need of kidney transplantation (KTx) typically spend a longer time waiting for compatible kidneys, are unlikely to receive an organ offer, and are at increased risk of antibody-mediated rejection (AMR). Desensitization using imlifidase, which is more rapid and removes total body immunoglobulin G (IgG) to a greater extent than other methods, enables transplantation to occur between HLA-incompatible (HLAi) donor–recipient pairs and allows patients to have greater access to KTx. However, when the project was launched there was limited data and clinical experience with desensitization in general and with imlifidase specifically. Hence, this Delphi methodology was used to reach a consensus from a multi-disciplinary team (MDT) of experts from 15 countries on the management of HS patients undergoing imlifidase HLAi from a deceased donor (DD) KTx. This Delphi consensus provides clinical practice guidance on the use of imlifidase in the end-to-end management of HS patients undergoing an HLAi DD KTx and supports centers in the development of guidelines for the utilization and integration of imlifidase into clinical practice.

## Graphical Abstract

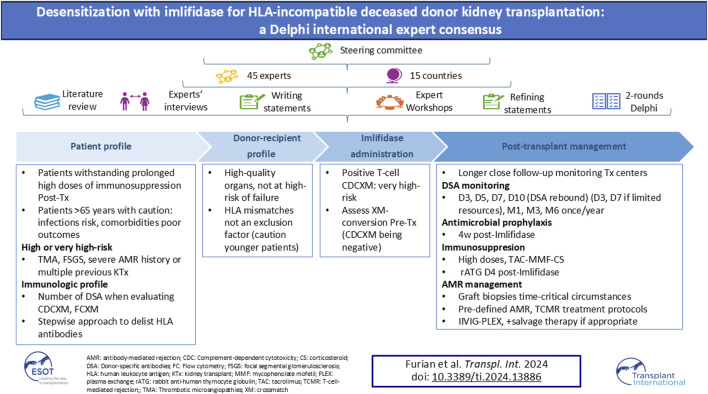

## Introduction

Sensitized patients with preformed human leukocyte antigen (HLA) antibodies, still face a curious situation, with longer waiting times and higher rejection risks [1–5]. Up to one-third of KTx candidates are sensitized [6], accumulating on waiting lists despite priority allocation programs [6–9]. The definition of HS may vary between countries and allocated regions [10], and patients wait longer for KTx and have higher AMR risks [1–5].

Worldwide, 5%–15% of patients are HS (panel reactive antibodies [cPRAs] ≥85%) [6, 7, 9, 11] and struggle to find compatible donors [8, 12, 13]. There is an increasing number of HS patients waitlisted worldwide with limited access to transplantation [14]. In Europe, Eurotransplant Kidney Allocation System data show that transplantation rates decrease as virtual panel reactive antibodies (vPRA) scores rise: 23% lower for scores ≤50%, 51% for 75%–85%, 65% for >85–95%, and 94% for 99%–100% compared with unsensitized candidates [1]. In the US, 2024 OPTN data showed that 11% of waiting for KTx candidates are HS (cPRA >80%, only 5% cPRA>98%), and 45% show some sensitization (at least cPRA >1%) [14]. Despite prioritization efforts in allocation programs in Europe and the U.S., 35% of HS patients rarely find compatible donors [15].

For HS KTx candidates, advances in desensitization have helped to enable transplantation mainly from living donors [16–18], although there are no drugs formally approved for this indication. Furthermore, protocols are often center-specific and comparisons between them are difficult. The preferred option for HS patients is to receive a compatible transplant through available kidney allocation systems, including prioritization programs [9, 14].

However, there is still a population of HS patients who are either not served or not eligible by prioritization programs who remain on waiting lists and for whom novel desensitization therapies are needed [1, 9].

Imlifidase (Idefirix^®^) is a cysteine proteinase derived from the IgG-degrading enzyme of *Streptococcus pyogenes* (IdeS) that cleaves IgG into F(ab′)2 and Fc fragments, inhibiting complement-dependent cytotoxicity (CDC) and antibody-dependent cellular cytotoxicity (ADCC) within hours [19], converting positive cross matches to negative, avoiding hyperacute rejection and enabling HLAi transplantation and [20–23], completely removing within hours total body IgG. It is well tolerated.

Imlifidase is conditionally approved by the EMA for desensitization before DD KTx, allowing patients to have greater access to KTx [22]. The reported 3 and 5-year data on Imlifidase HLAi KTx [15], showed positive outcomes with 90% patient and graft survival (death censored) of 84% and 82% at 3 and 5 years respectively [11, 15, 23]. The ESOT ENGAGE initiative reported consensus for imlifidase as a desensitization strategy for DD KTx in highly selected patients with no other options [14]. Although imlifidase is a potent option for overcoming significant immunologic barriers, data and clinical experience with desensitization and imlifidase specifically, remain limited, with countries developing their own consensus guidelines on its use [16, 24].

### Aims

To consolidate expert opinion on the evaluation and management of HS patients undergoing HLAi KTx from DD after imlifidase desensitization and to guide transplant physicians in identifying and managing these patients and integrating imlifidase into their center’s protocols.

## Materials and Methods

The international expert panel consisted of 45 European and U.S. transplant nephrologists, surgeons and HLA specialists. Experts were selected based on imlifidase experience or expertise in the field of KTx and/or HLAi transplantation and AMR management.

An iterative approach was developed to reach consensus, following a series of qualitative and quantitative methods based on the Accurate Consensus Reporting Document (ACCORD) guidelines [25], summarized in Table 1.

**TABLE 1:** Iterative approach to reaching a consensus on a series of statements.

Step	Description
1	To identify a **multidisciplinary Steering Committee** to lead and coordinate the guideline development process
2	To **identify the key topics** involved in the transplant physician’s decision-making process when evaluating and managing a highly sensitized patient for an HLAi KTx from a DD with imlifidase
3	**Literature review** to identify the current body of research and the major gaps and inconsistencies in the HLAi KTx clinical practice guidelines
4	**Interviews** with three experts to explore and challenge initial assumptions
5	**The Steering Committee meeting** to discuss experts’ views on three predefined risk categories of highly sensitized patients (moderate, high, and very-high risk) was explored
6	**Interviews** with three additional experts to refine and validate the outputs and assumptions from the Steering Committee
7	**First pan-EU Expert Workshop** with 45 expert participants from Europe and the USA to discuss and test these outputs and assumptions. This provided a broader first view of the level of consensus that started to be built on key topics and considerations in the clinical decision-making and risk stratification process of transplant physicians during HLAi KTx
8	Analysis of the insights from the pan-EU Expert Workshop and consolidation into discrete “**expert opinion statements**”. A framework for the initial list of statements was defined, enabling structured thinking and the involvement of experts in their areas of expertise
9	**Nine 1-h Expert Review Sessions** in which experts further updated and refined the expert opinion statements in an iterative manner. This culminated in the third iteration of the Imlifidase Clinical Workbook, which consisted of refined expert opinion statements and open-ended questions based on feedback from all experts
10	Finally, these statements were evaluated and responded to in the next phase of the project using a **Delphi methodology with two rounds** of surveys • Following the first round of surveys, the results were analyzed, and the statements and questions were prioritized for discussion during the second Pan-EU Expert Workshop; the prioritization was based on the level of discrepancy and disagreement among panelists, with the aim of challenging and further validating expert consensus and non-consensus. The outputs were used to update and finalize the expert opinion statements which were tested again in the second round of surveys. In this second round, experts had the opportunity to compare their own initial responses and reconsider agreement levels based on the group response from the first round of surveys
11	A thorough qualitative and quantitative analysis of the responses from the second survey was conducted, which ultimately informed the **final content** and respective level of consensus of all the expert opinions

Bold text was simply to facilitate the reading.

### Delphi Methodology

**FIGURE 1:**
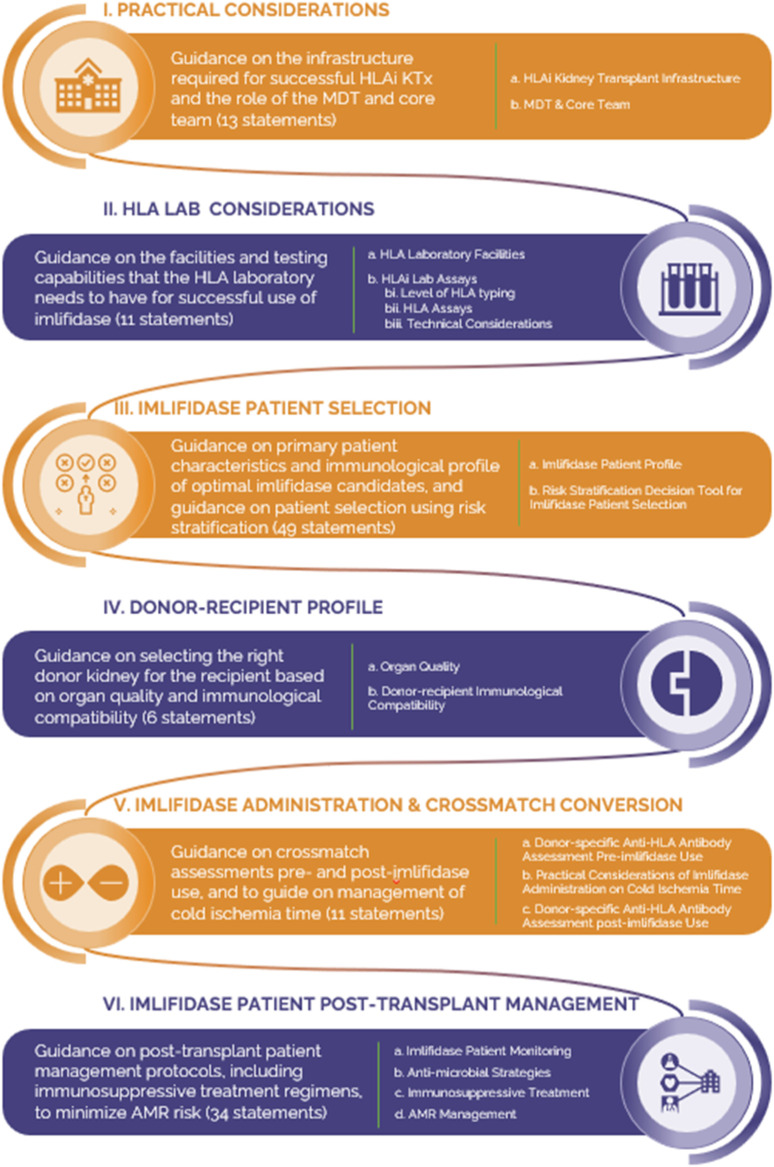
The six sections of the Delphi questionnaire that evaluated the various aspects of kidney transplantation and the utilization of imlifidase.

The Delphi methodology [26, 27] was employed to gather global insights on managing HS patients receiving imlifidase HLAi KTx. It was performed in May 2022, when only 46 clinical trial patients were treated with imlifidase, mostly in the U.S. and Sweden. The questionnaire included six sections on imlifidase KTx (see Figure 1).

The online survey was completed in two rounds. In the first round, experts voted on the degree of agreement with each statement using a 5-point Likert scale (1 = strongly disagree; 2 = disagree; 3 = neutral; 4 = agree; 5 = strongly agree). Statements reaching ≥75% agreement were considered consensual, while for others, members explained their disagreement.

Statements with lack of agreement were re-written and clarified by the expert panel and re-evaluated in the second round. The results show the percentage of agreement for each final statement after the two rounds.

## Results

The consensus statements representing the opinions of the 45 experts from 15 countries who participated in the modified Delphi study are gathered in Supplementary Tables S1-S5, with their corresponding levels of agreement.

## Discussion

### HLAi KTx Infrastructure and Team Resources

There was broad consensus on the need for an optimal infrastructure and MDT to initiate an HLAi KTx program in a transplant center. DD HLAi KTx protocols should be in place for organ retrieval, equitable organ allocation and organ preservation, together with appropriate imlifidase protocols to facilitate transplantation for HS patients who might otherwise be considered unsuitable (87.5% consensus) (Supplementary Table S1).

It is advised that an integrated approach among centers be taken with DD HLAi KTx (90.6% consensus) and referring nephrologists and dialysis centers should be informed about the possibility of imlifidase HLAi KTx so that potential patients can be referred to an HLAi KTx expert center to further evaluate their eligibility (90.6% consensus) (Supplementary Table S1).

Experts advised that centers should have 24/7 access to HLA laboratory services to address the need for close monitoring of HS patients potentially undergoing HLAi KTx (93.8% consensus) (Supplementary Table S1). Indeed, when considering imlifidase HLAi KTx, access to an HLA laboratory is considered essential for the appropriate selection of donor–recipient pairs [6, 28] Assessment of a potential recipient’s sensitization history and degree of HLA mismatch with the DD is critical prior to accepting an offer [29]. Post transplantation, appropriate patient monitoring including access to an HLA laboratory allows for monitoring of donor-specific antibodies (DSA), renal function assessment and graft biopsy, to diagnose early AMR and initiate appropriate treatment as soon as possible [30]. According to a clinical study, DSA rebound following imlifidase occurs in 80% of the patients at 3–14 days post-treatment [20]. Hence, immediate access to HLA assessment facilities is critical for effective patient management.

A multidisciplinary approach is advised for evaluating patients’ physiological status (87.1% consensus). Similarly, an MDT comprising transplant surgeons, nephrologists, HLA specialists, transplant coordinators, pathologists, specialized nurses, pharmacists, and ICU specialists should be established to evaluate patient eligibility and progress with HLAi KTx (90.6% consensus), and MDT members should be trained and prepared for imlifidase HLAi KTx, including awareness of center-specific patient management protocols and procedures (87.5% consensus) (Supplementary Table S1). Furthermore, experts advised that a dedicated HLAi KTx imlifidase expert core team (comprising a transplant surgeon, nephrologist, and HLA specialist) be in place and available 24/7 in the case an offer occurs (93.6% consensus) (Supplementary Table S1). This core team of experts would advise on key decisions regarding patient eligibility and management, particularly when evaluating and approving organ suitability at the time of the offer (96.8% consensus).

There was 100% consensus that a multidisciplinary approach should be taken in the case of an HLAi donor offer to assess the individual patient (immunological) risk that a pre-formed DSA might pose and to ensure appropriate management when the donor offer comes in (Supplementary Table S1).

Experts also recommended that the MDT dedicate sufficient time to educate potential imlifidase patients on the risks and adherence requirements prior to HLAi KTx and throughout the process (96.9% consensus) (Supplementary Table S1). This is likely to require several sessions as the majority of these patients are on long-term dialysis and are not expecting transplantation to be an option, therefore they have to adjust to this to evaluate the risk–benefit of treatment and post-transplantation immunosuppressive therapy [28]. Long-term immunosuppression carries risks of adverse events [31] that patients need to be aware of, although many have previous experience with immunotherapy, together with the importance of treatment adherence to improve long-term outcomes and long-term tacrolimus and mycophenolic acid exposure target levels to prevent rejection [32].

At the first use of imlifidase, experts advised treating one patient at a time. This would enable the practical application of HLAi KTx processes into clinical practice (87.5% consensus) (Supplementary Table S1), which is likely to increase the chance of successful transplantation, build the experience of the MDT at the center and allow amendment of any protocols should it be necessary.

### HLA Laboratory Facilities and Assays

Focusing on technical support/facilities within the transplant centers, the laboratory/testing facilities should have rapid turnaround times particularly for crossmatch evaluation to limit organ cold ischemia time (CIT) (100% consensus) (Supplementary Table S2). Furthermore, crossmatch conversion from positive to negative in patients treated with imlifidase should be confirmed before transplantation [23]; therefore, in addition to having HLA assessment facilities, rapid assay turnaround times are also important when performing an imlifidase transplant to keep CIT as short as possible because CIT impacts kidney graft survival rates [33]. To increase this speed, some centers are deciding to transplant based on virtual crossmatch conversion, i.e., single-antigen bead (SAB) data showing a significant decrease in DSA with FCXM as a retrospective test.

Experts advised that HLA typing at the resolution of the recipient or donor profile is sufficient to determine compatibility for each case, preferably typing for all 11 HLA loci (HLA-A, HLA-B, HLA-C, DPA1, DPB1, DQA1, DQB1, DRB1, DRB3, DRB4, and DRB5) (90.6% consensus) (Supplementary Table S2). It was also recommended that allelic, high-resolution typing be performed whenever possible (93.6% consensus) and that this should become the future standard for all HS patients (90.6% consensus) (Supplementary Table S2).

Experts advised that HLA laboratories follow a method of serum treatment for all HS patient samples to reduce complement interference (93.3% consensus) and non-complement-mediated prozone effects to improve accurate HLA antibody detection (87.1%) (Supplementary Table S2). Technical issues impact single antigen assays and may confound assay interpretation. For example, false negative results may occur due to complement interference. Prozone is reportedly very frequent in HS patients (87%), particularly in those with a history of previous transplantation [34].

In the first few (<4) hours post-imlifidase administration, experts advised against the use of an Fc-detecting antibody-based SAB assay as this can lead to false positive signals due to the high amount of single-cleaved IgG (80% consensus) (Supplementary Table S2). As other treatments used in conjunction with imlifidase may also interfere with assay results, experts advised that post-imlifidase HLAi KTx, potential effects of intravenous immunoglobulin (IVIg), rabbit anti-human thymocyte globulin (rATG) or anti-CD20 mAb (rituximab) on assay results should be considered (86.7% consensus) (Supplementary Table S2).

### Primary Characteristics of the Imlifidase Patient Profile

#### Primary Patient Characteristics

Experts recognized the importance of selecting only those HS patients who are considered capable of tolerating prolonged high doses of immunosuppression following transplantation (88.9% consensus) (Supplementary Table S3) since imlifidase administration does not reduce the immunosuppressive burden required in HLAi KTx both in terms of induction and maintenance therapy.

Patient characteristics such as comorbidity, primary renal disease, immunological risk, dialysis/previous transplant history and psychosocial factors may influence the potential outcomes of HLAi KTx [35]. Older patients may be more susceptible to infection following KTx [36] and more likely to have comorbidities. While experts advised that chronological age should not be restrictive and that patients should be considered primarily based on their physiological age in the context of other comorbidities (88.9% consensus), they also advised that patients older than 65 years should be approached with extra caution considering the higher risk of infection and poor outcomes associated with this age group (75% consensus) (Supplementary Table S3). The assessment and risk stratification of HS patients has become even more challenging as the number of transplant recipients over 60 years of age increases resulting in an increased incidence of comorbidities contributing to kidney failure, such as diabetes, hypertension, and obesity [37].

Often associated with age is frailty, and while experts advised that patient frailty status be assessed by the MDT and should include physical and cognitive evaluation (88.6% consensus), consensus was not reached (61.1%) on whether a validated frailty score should be developed specifically for HS patients, given the complexity and higher HLAi KTx risk and lack of standardized frailty evaluation across centers (Supplementary Table S3).

Experts advised considering patients with an expected survival rate of ≥5 years unless there are pressing reasons for transplantation or a significantly high unmet need (90.6% consensus) (Supplementary Table S3). Other characteristics to be considered when stratifying patients as being at high or very high risk that were confirmed and highlighted by experts here include thrombotic microangiopathy (75%) and primary focal segmental glomerulosclerosis (FSGS) (83.3% consensus) (Supplementary Table S3). However, no consensus was reached on original kidney disease with a high recurrence risk as a (relative) contraindication for HLAi KTx (71% consensus). HS patients with severe AMR history (84.4%) or multiple previous KTx should be considered at high risk for AMR after HLAi KTx (90.6% consensus), while patients who have exhausted standard routes of vascular access are at high risk for adverse outcomes on dialysis and should be prioritized for an HLAi KTx (80.6% consensus) (Supplementary Table S3).

#### Patient Immunological Profile

Experts advised conducting HLA antibody screening using SAB for all HS patients at regular intervals according to national and local guidelines, preferably every 3 months, and after 2-3 weeks following desensitization and immunization events (94.4% consensus).

In addition, historical DSA data and screening for circulating preformed anti-HLA specific antibodies should be part of the pre-transplant immunological risk assessment for all HS patients (100% consensus) (Supplementary Table S3). Furthermore, considering the different protocols and assays across countries and transplant centers, it was advised that each center has its own reference values to estimate the likelihood of rejection (93.8% consensus) (Supplementary Table S3).

Similarly, when assessing a patient’s sensitization level, it is important to integrate the strength of the antibody response assessed using mean fluorescence intensity (MFI) in undiluted serum, the breadth of sensitization (assessed using cPRA) and the specificities to create an immunological risk profile.

#### DSA Characteristics

It was explored whether patient sera should be treated appropriately according to local laboratory protocols when assessing DSA strength to ensure prozone effect inhibition. There was consensus regarding the use of ethylenediaminetetraacetic acid (EDTA) treatment (83.9% consensus) but not on serial dilutions (61.3%) or heat activation (45.2%) (Supplementary Table S3).

Despite these results, serial dilutions have been reported to help estimate true cPRA in HS candidates and in evaluating DSA strength. Furthermore, pretransplant serum dilutions can be used to determine unacceptable antigens, and the likelihood of successful HLA antibody reduction with desensitization [24].

Antibody specificities should be confirmed using a physical crossmatch assay to prevent considering non-relevant antibodies directed against denatured HLA as a risk. When discussing DSA strength in terms of MFI value, the following thresholds were used as guidance for the discussion: <3,000 – low; 3,000–5,000 – intermediate; 5,000–10,000 – high; and >10,000 – very high clinical significance and immunological risk.

Delisting unacceptable antigens that are considered lower risk allows transplant physicians to amend a patient’s profile within reasonable limits, removing barriers to receiving a transplant despite immunological incompatibilities [38]. When delisting is permitted by the allocating organization, experts have recommended a stepwise approach to delisting as many unacceptable HLA antigens as deemed appropriate according to these parameters: a) start with delisting unacceptable HLA antigens with low-risk DSA (MFI values < 3,000, never crossmatch positive) and then proceed with delisting unacceptable HLA antigens for DSA with intermediate MFI values; b) avoid delisting unacceptable HLA antigens for repeated mismatches and for DSA with a historically positive crossmatch or C1q or C3d assay taking into account memory B cells; and c) take into consideration the additional contributing risk factors when assessing the antibody titers and potential post-transplant rebound risk (83.9% consensus) (Supplementary Table S3).

### Donor–Recipient Profile

#### Organ Quality

Focusing on DD kidneys, experts advised selecting high-quality organs that are not at high risk of failure (no signs of severe acute tubular necrosis or acute kidney injury) unless there are pressing reasons to consider otherwise (77.8% consensus), and that organ quality and function be validated by the recipient transplant center administering imlifidase (88.9% consensus) (Supplementary Table S4).

For successful long-term transplant outcomes irrespective of the patient’s degree of sensitization, it is critical to begin with good organ quality. A donor’s kidney needs to have sufficient nephron mass to meet the increased and long-term metabolic demands and stress that a single kidney will incur in the recipient [39]. Kidneys at high delayed graft function risk and with a reduced functional reserve will have a more negative impact in this population of patients [40]. In addition, delayed graft function will also make rebound DSA and AMR assessment more complicated as no clinical parameters of renal function or laboratory values can be followed during this time period. Hence, assessment of kidney quality is critical at the time of transplantation, particularly in donors with suboptimal conditions (older age, uncertain medical history, pre-donation renal failure) [39].

#### Donor–Recipient Immunological Profile

As advised by experts, HLA polymorphism poses a significant risk in transplantation due to incompatible HLA profiles between recipient and donor (86.1% consensus) (Supplementary Table S4), and the greater the disparity in HLA the greater the risk of graft failure regardless of the presence of DSA prior to transplantation [41]. Experts also advised that the number of HLA mismatches should not be an exclusion factor for accepting a donor’s kidney, provided there is sufficient prior experience with HLAi transplants (86.1% consensus), although whenever possible it is advised to aim for fewer mismatches in younger recipients due to their potential need for future transplant(s) (86.1% consensus) (Supplementary Table S4).

### Imlifidase Administration and Crossmatch Conversion

As mentioned, before Imlifidase administration, experts advised that donor–recipient immunological compatibility be assessed according to the local laboratory protocols and that at least one flow cytometric-crossmatch (FCXM) or a CDC-crossmatch (CDCXM) be performed paired with a fresh or recent (<6 weeks) SAB assay (83.9% consensus) (Supplementary Table S5).

Such data will provide more assurance around risk assessment and generate evidence to further support risk stratification and interpretation across patients. Experts advised that each center has pre-defined criteria for assessing FCXM as borderline positive, clearly positive or very positive. It is advised that HLAi KTx with *borderline positive FCXM* undergo transplantation with or without imlifidase, but post-transplant management with higher levels of immunosuppression compared with FCXM negative HLAi KTx; *clearly positive FCXM* be considered to be at high immunological risk and treated using imlifidase; *very positive FCXM* (positive CDCXM) be considered to be at very high immunological risk and either not proceed with the transplant or be treated with imlifidase, provided there are significant pressing reasons and prior experience with HLAi KTx (77.4% consensus) (Supplementary Table S5). This is consistent with the agreement reached by the ENGAGE Delphi consensus, where experts agreed that imlifidase could be considered as a desensitization strategy for DD KTx in patients with positive CDCXM or patients with positive FCXM at day 0 who have no other treatment options.^25of^

Provided there is sufficient time and donor/recipient cells, experts advised crossmatch conversion assessment via a physical crossmatch (CDCXM or FCXM), after a second dose of imlifidase according to local practice before proceeding with transplantation (82.7% consensus) (Supplementary Table S5).

In patients treated with imlifidase, CDCXM conversion from positive to negative should be confirmed before transplantation [23]. It should be noted that consensus was not reached on a second dose of imlifidase being administered within 24 h of the first dose if the crossmatch had not been converted (71% consensus) (Supplementary Table S5), despite this being within the product label [23].

### Post-Transplant Management, Monitoring and Follow-Up of Imlifidase Patients

Experts recommended that patients be kept at the transplant center for as long as possible immediately following HLAi KTx to ensure close monitoring is conducted and optimal care is provided during the first 10–15 days (75% consensus), and that open communication channels be established between the hospital and transplant center (should they be separate) to ensure best practice protocols are in place for post-transplant management and emergency response (87.5% consensus) (Supplementary Table S6).

It is also advised that monitoring of kidney function, infections and overall clinical status of the patients post-transplantation be conducted in line with local and national guidelines (97.1% consensus) (Supplementary Table S6). Longer-term follow-up post-HLAi KTx is also advised, and patients should visit the transplant center at regular intervals following their transplant, preferably at least: twice a week for the first 1–2 months; twice a month for the following 3–4 months; once (stable patients) or twice a month (patients at higher risk of AMR) for the following 6 months; and once a year after this (87.1% consensus), although initially every 3 months may be more appropriate (Supplementary Table S6).

#### DSA Monitoring

Experts recommended close monitoring of DSA using an SAB assay to increase the likelihood of identifying DSA rebound (93.8% consensus) or antibody rebound (93.8% consensus) as close to the time of occurrence as possible (Supplementary Table S6). The aim is to ensure early identification of AMR and that treatment to prevent chronic AMR is initiated in a timely manner. It is recommended to assess DSA following the transplant on Days 3, 5, 7, and 10 (not if IVIG is given on Days 9 and 10); Months 1, 3 and 6; and then once a year (87.1% consensus) (Supplementary Table S6).

Experts also advised considering the potential interfering effect of IVIg on SAB assay results and adapting the frequency of DSA monitoring accordingly (81.3% consensus) (Supplementary Table S6).

#### Antimicrobial Prophylaxis

Experts advised that antimicrobial prophylaxis be provided to all patients prior to and following HLAi KTx, according to local protocols and individual patient risk factors (96.8% consensus), and that antimicrobial prophylaxis be maintained for at least 4 weeks post-imlifidase transplantation (77.4% consensus) (Supplementary Table S6).

It is also advised that all patients receive vaccination against infections such as influenza, pneumonia, and COVID-19 before imlifidase treatment, and at least 2 weeks apart from any cell-depleting therapy (100% consensus) (Supplementary Table S6). These strategies align with protection against infections that may occur because of the long-term immunosuppression that is required post-transplantation to prevent graft rejection. Imlifidase temporarily reduces IgG levels (hypogammaglobulinemia), and the most common infections associated with this are respiratory tract infections. Therefore, in addition to the standard antimicrobial prophylaxis in KTx (*Pneumocystis carinii*, cytomegalovirus and oral *candida*), imlifidase patients may require antimicrobials to treat respiratory tract pathogens [23]. Should a patient for any reason not be transplanted after receiving imlifidase treatment, prophylactic oral antimicrobials should still be given for 4 weeks [23].

#### Immunosuppressive Therapy

It is advised that the induction and maintenance IS protocol be tailored to the needs of HS patients (93.6% consensus), that steroids be used in all patients regardless of risk profile and that early withdrawal of steroids be avoided (94.5% consensus) (Supplementary Table S6).

It is advised that high doses of immunosuppression, preferably a triple-agent regimen (tacrolimus, mycophenolate and corticosteroid), be provided to all patients according to local protocols and their individual risk factor needs (94.4% consensus), and that calcineurin inhibitors (100% consensus) and IMDH inhibitors (e.g., MMF) be considered as part of the immunosuppression regimen according to standard of care (SoC) protocols (91.7% consensus) (Supplementary Table S6).

#### AMR Management

Should acute graft rejection occur, it may be T-cell-mediated rejection (TCMR), AMR or both [42]. Confirmation of AMR is provided by kidney biopsy and the presence of microvascular inflammation, an accumulation of inflammatory cells in the graft capillaries (glomerulitis and/or peritubular capillaritis ≥2), with or without the presence of deposits of the complement fraction C4d in the peritubular capillaries, and with circulating DSA against donor HLA antigens [42, 43]. In centers where molecular assessment is available its utilization to detect early stages of AMR, especially early after HLAi KTx, would be beneficial. Experts advised that plasmapheresis should be considered as part of the SoC protocols for AMR management and that the patient’s individual risk factor should be assessed (93.8% consensus). Experts also advised that any arising immunological complications should be managed exclusively by the transplant center regardless of the time passed since the HLAi KTx (86.1% consensus) (Supplementary Table S6).

Experts advised that predetermined protocols for the treatment of AMR (91.7%) or TCMR (94.5% consensus), acute and chronic, should be well defined in advance and in place for Imlifidase KTx, according to national and local guidelines, to ensure an immediate clinical response can occur (Supplementary Table S6). Biopsies should be performed in time-critical circumstances and cases of severely impaired renal function and suspected AMR anti-rejection treatment should be initiated directly, prior to performing or receiving results from a biopsy (96.8% consensus) (Supplementary Table S6). Experts also advised that AMR management should follow local AMR protocols but be implemented earlier and with a more rapid stepwise approach, including earlier initiation of a complement inhibitor if needed. If AMR is still not appropriately managed, it is advised to consider alternative options such as splenectomy (87.1% consensus) (Supplementary Table S6) or targeting plasma cells in refractory patients.

## Conclusion

**FIGURE 2:**
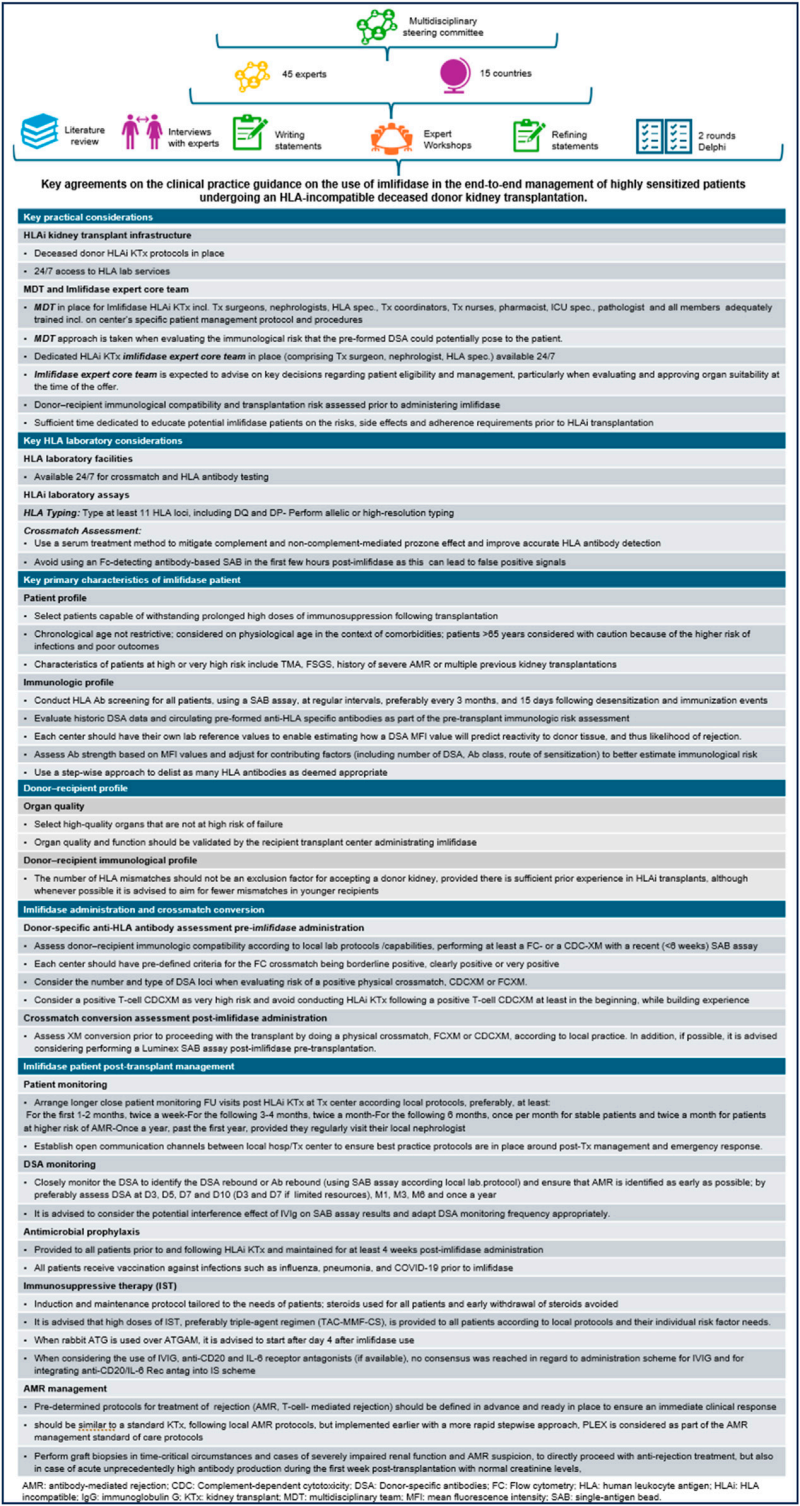
Visual abstract.

HS patients in need of KTx spend a longer time waiting for compatible kidneys and are often unlikely to receive them. Imlifidase desensitization, which is more rapid and removes total body IgG to a greater extent than other methods, may offer a unique opportunity, especially for DD transplantation, to significantly reduce, albeit only transiently, the risk of hyperacute and accelerated graft rejection and may provide access to transplantation [14, 22, 23]. This Delphi consensus provides clinical practice guidance on Imlifidase use in the management of HS patients undergoing HLAi DD KTx and supports centers in the development of guidelines for imlifidase use and its integration into clinical practice (Figure 2). Due to the limited data available at the time of the development of this study and the subsequent uncertainty about the use of imlifidase for desensitization for KTx, increasing clinical experience will further refine the therapeutic guidelines.

## Data Availability

The original contributions presented in the study are included in the article/Supplementary Material, further inquiries can be directed to the corresponding author.

## Abbreviations

ACCORD, Accurate Consensus Reporting Document; ADCC, antibody-dependent cellular cytotoxicity; AMR, antibody-mediated rejection; CDC, complement-dependent cytotoxicity; CDCXM, complement-dependent cytotoxicity crossmatch; CIT, organ cold ischemia time; cPRA, calculated panel reactive antibody; DD, deceased donor; EDTA, ethylenediaminetetraacetic acid; ESOT, European Society for Organ Transplantation; FC, flow cytometric; FCXM, flow cytometric crossmatch; FSGS, focal segmental glomerulosclerosis; HLA, human leukocyte antigen; HLAi KTx, HLA-incompatible kidney transplantation; HS, highly sensitized; IdeS, IgG-degrading enzyme of *S. pyogenes*; IgG, immunoglobulin G; IVIg, intravenous immunoglobulin; IS, immunosuppressive; KTx, kidney transplantation; MDT, multidisciplinary team; rATG, rabbit anti-human thymocyte globulin; SAB, single-antigen bead; SoC, standard of care; TCMR, T-cell-mediated rejection; vPRA, virtual panel reactive antibodies.
