# Supplementary material for: Desensitization With Imlifidase for HLA-Incompatible Deceased Donor Kidney Transplantation: A Delphi International Expert Consensus
Source: Transpl Int. 2025 Jan 6;37:13886. doi: 10.3389/ti.2024.13886 (PMC11758882; doi:10.3389/ti.2024.13886)
Supplement: Supplementary file 1 [file DataSheet1.docx]

**Supplementary material**

**Supplementary Table S1.** Statements and level of agreement on HLAi Kidney transplant infrastructure and human resources team.

| **Statement** | **% of agreement** |
| --- | --- |
| **HLAi Kidney transplant infrastructure** |  |
| It is advised that deceased donor HLAi KTx protocols be in place for organ retrieval, equitable organ allocation, organ preservation, evaluation of brain death, and seeking of consent from relatives. | 87.50% |
| It is advised that an integrated approach be taken with deceased donor HLAi KTx, with well-established referral patterns from nephology centers, cooperation and involvement of intensive-care unit (ICU) and high-dependency unit (HDU) specialists as well as organ procurement teams and HLA laboratories that are ideally available 24 hours a day. | 90.60% |
| It is advised that referral nephrologists and dialysis centers be informed about the possibility of HLAi KTx with imlifidase and that they could refer their potential patient for further eligibility evaluation to an HLAi KTx expert center. | 90.60% |
| It is advised that centers have 24/7 access to HLA lab services in order to be able to address the close monitoring needs of highly sensitized patients and HLAi KTx. | 93.80% |
| **Multidisciplinary team (MDT) and Core team** |  |
| A multidisciplinary approach is advised for evaluating patients’ physiological status. | 87.10% |
| It is advised that an MDT be in place to evaluate patients’ eligibility for and progress in HLAi KTx, consisting of the following specialists: transplant surgeons, nephrologists, HLA specialists, transplant coordinators, pathologists, specialized nurses, pharmacist, ICU specialists. | 90.60% |
| It is advised that all MDT members be trained and prepared for HLAi KTx, including being aware of center-specific patient management protocols and procedures to support the expert core team. | 87.50% |
| It is advised that a dedicated HLAi KTx expert core team be in place that would be responsible for managing individual patients and be expected to be available 24/7 in case an organ match occurs, consisting of the following specialists: transplant surgeon(s), nephrologist(s), HLA specialist(s). | 93.60% |
| The expert core team will be expected to advise on key decisions regarding patient eligibility and management, particularly when evaluating and approving organ suitability at the time of the offer. | 96.80% |
| It is advised that a multidisciplinary approach is taken when evaluating the immunological risk that the pre-formed DSA could potentially pose to the patient. | 100% |
| At the time of the offer, it is advised that the donor-recipient immunological compatibility and transplantation risk be assessed by the core team prior to administering imlifidase to ensure clinically informed and quick action is undertaken. | 96.90% |
| It is advised that the MDT and expert core team dedicate sufficient time to educate potential imlifidase patients on the risks, side effects and adherence requirements prior to HLAi transplantation as well as regularly throughout the process. | 96.90% |
| At first use of imlifidase, it is advised treating one patient at a time in order to enable practical application of HLAi KTx processes into clinical practice. | 87.50% |

DSA: donor-specific antibody; HLA: human leukocyte antigen; HLAi: HLA incompatible; ICU: intensive care unit; KTx: kidney transplant; MDT: multidisciplinary team.

**Supplementary Table S2.** Statements and level of agreement regarding HLA laboratory facilities and HLAi laboratory assays.

| **Statement** | **% of agreement** |
| --- | --- |
| **HLA laboratory facilities** |  |
| It is advised that centers ideally have 24/7/365 access to HLA lab services in close proximity in order to be able to address the frequent assessment and monitoring needs of patients undergoing an HLAi KTx. | 93.80% |
| It is advised that centers work closely with their designated HLA lab to align on testing turnaround to mitigate the prolongation of timelines, particularly for organ CIT and crossmatch evaluation. | 100% |
| **HLAi laboratory assays** |  |
| ***HLA typing*** |  |
| It is advised that the level of typing resolution when assessing the HLA profile of the recipient or donor be sufficient to determine compatibility for each case, preferably typing at least 11 HLA loci, including DQ and DP. | 90.60% |
| For ultimate comprehensive compatibility assessment, it is advised to perform allelic or high resolution typing whenever possible. | 93.60% |
| It is advised that high resolution typing be the future standard in tissue typing for all HS patients, considering the immunological complexity and higher risk of HLAi KTx. | 90.60% |
| ***Crossmatch assessment*** |  |
| It is advised that HLA laboratories follow a method of serum treatment for all HS patient samples in order to mitigate **complement-mediated** prozone effect and improve accurate HLA antibody detection. | 93.30% |
| It is advised that HLA laboratories follow a method of serum treatment for all HS patient samples in order to mitigate **non-complement-mediated** prozone effect and improve accurate HLA antibody detection | 87.10% |
| Within the first few hours post-imlifidase administration, it is advised to avoid using an Fc-detecting antibody-based SAB as this can lead to false positive signals due to the high amount of intact IgG and sclgG. | 80% |
| Following HLAi KTx with imlifidase, it is advised to consider the potential effect of IVIg, rATG and anti-CD20 mAb (rituximab) on assay results, and thus adapt the timing of the assay accordingly to evaluate patients’ HLA Ab after administering IVIg or anti-CD20 mAb. | 86.70% |
| For patients who are treated with rituximab prior to transplantation, it is advised to consider the potential effect of rituximab on CDC and FC crossmatch assay results and thus consider treating sera with high concentrations (>1mg/ml) of pronase and or anti-rituximab mAb | 86.70% |
| For patients who are treated with rituximab and have been administered imlifidase, it is advised to consider the potential effect of rituximab and imlifidase on CDCXM assay results and thus consider treating sera with pronase or anti-rituximab Ab and anti-human globulin directed against the Fc-portion and not against the Fab-portion of the IgG | 67.90% |

Ab: antibody; CIT: cold ischemia time; HLA: human leukocyte antigen; HLAi: HLA incompatible; HS: highly sensitized; IgG: immunoglobulin G; IVIg: intravenous immunoglobulin; KTx: kidney transplant; mAb: monoclonal antibody; rATG: rabbit anti-human thymocyte globulin; SAB: single-antigen bead; sclgG: single-cleaved immunoglobulin G

**Supplementary Table S3.** Statements and level of agreement on primary characteristics of imlifidase patient profile.

| **Statement** | **% of agreement** |
| --- | --- |
| **Imlifidase Patient Profile: Primary Patient Characteristics** | |
| It is advised that patients above the age of 65 be approached with extra caution considering the higher risk of infections and poor outcomes associated with this age group. | 75% |
| It is advised that chronological age not be restrictive, and patients be considered primarily based on their physiological age in the context of other comorbidities. | 88.90% |
| It is advised selecting patients that are considered capable of withstanding prolonged high doses of immunosuppression following transplantation. | 88.90% |
| It is advised considering patients with expected survival rate of 5 years or more unless there are pressing reasons for transplant or significantly high unmet need. | 90.60% |
| It is advised that the patient’s frailty status be assessed from an MDT, including physical and psychological evaluation. | 88.60% |
| It is advised that a validated frailty score tool be developed specifically for HS patients, considering the complexity and higher risk of HLAi KTx and lack of standardized frailty evaluation across centers. | 61.10% |
| It was also explored which of the following patients should be considered for an HLAi kidney transplant: preferably patients with expected survival of over 5 years; all patients regardless of their expected survival rate; preferably patients with expected survival rate of over 5 years or of fewer than 5 years if there are pressing reasons for transplant or significantly high unmet need.   - Preferably patients with expected survival of over 5 years should be considered for an HLAi kidney transplant. - All patients regardless of their expected survival rate should be considered for an HLAi kidney transplant. - Preferably patients with expected survival rate of over 5 years or of fewer than 5 years if there are pressing reasons for transplant or significantly high unmet need should be considered for an HLAi kidney transplant. |  |
|  | 45.10% |
|  | 9.70% |
|  | 45.10% |
| It is advised that patients with lupus nephritis be considered as being at intermediate risk. | 30.60% |
| It is advised dedicating time for patient consultations and education to explain the higher risk associated with HLAi KTx and thoroughly discuss the patient’s appetite for risk, personality profile, as well as caregiver and social support status. | 100% |
| It is advised that integrated and multidisciplinary care include uniform criteria and procedures for standard assessments, for patient autonomy, adherence to therapy, new coping strategies and the adoption of more appropriate lifestyles. | 75% |
| **Imlifidase Patient Profile: Immunological Profile** |  |
| ***Anti-HLA antibody profile*** |  |
| It is advised conducting HLA antibody screening for all HS patients, using a solid-phase assay (Luminex Single-Antigen Beads) assay (SAB), at regular intervals according to national and local guidelines, preferably every 3 months, and 15 days following desensitization and immunization event. | 94.40% |
| It is advised evaluating historic DSA data and circulating pre-formed anti-HLA specific antibodies as part of the pre-transplant immunological risk assessment for all HS patients. | 100% |
| Considering the diverse protocols and assays across countries and transplant centers, it is advised that each center has their own lab reference values to enable estimating how a DSA MFI value will predict reactivity to donor tissue, and thus likelihood of rejection. | 93.80% |
| It is advised assessing the antibody strength based on MFI values and adjusting for contributing factors (including number of DSA, Ab Class, route of sensitization), to better estimate the patient’s immunological risk. | 96.90% |
| It is advised that the strength of a DSA of MFI value (MFI thresholds to be adapted based on local lab reference):   - <3000 be considered of potentially low clinical significance and immunological risk. - 3000–5000 be considered of potentially intermediate clinical significance and immunological risk. - 5000–10000 be considered of potentially high clinical significance and immunological risk. - >10000 be considered of potentially very high clinical significance and immunological risk. | 87.50% |
| It was explored whether the patient’s sera should be appropriately treated according to local lab protocols when assessing the strength of DSA in order to ensure inhibition of the prozone effect, preferably through:   - EDTA treatment. - Heat activation. - Serial dilutions. |  |
|  | 83.90% |
|  | 45.20% |
|  | 61.30% |
| ***Sensitization route*** |  |
| It is advised that the sensitization route be considered when determining the potential immunological risk posed by an individual DSA, along with other contributing factors. | 72.20% |
| ***Determining the permissive anti-HLA antibodies*** |  |
| In countries where the practice of delisting is permitted, it is advised that a step-wise approach is considered to delist as many HLA deemed appropriate according to the below parameters: | 83.90% |
| - Start with delisting HLA for DSA with low MFI values, then proceed with delisting HLA for DSA with intermediate MFI values. |  |
| - Avoid delisting HLA for repeated mismatches and for DSA with a historically positive CDCXM or C1q assay. - Take into consideration the additional contributing risk factors when assessing the Ab strength and potential post-transplant rebound risk |  |
| ***Other immunological considerations*** |  |
| It is advised considering assessing non-HLA antibodies against angiotensin type 1 (AT1) receptors or T-cell ELISPOT assay of alloantigen-specific donor, in patients with history of non-HLA Ab present from previous transplant(s) | 51.60% |
| It is advised, in addition to HLA Ab assessment, considering assessing for non-HLA autoantibodies and alloantibodies in patients with history of non-HLA Ab present from previous transplant(s) when determining the immunological risk of a patient. | 46.90% |
| For future research purposes, it is advised to consider assessing non-HLA antibodies in all HS patients. | 58.30% |
| ***Pre-emptive desensitization*** |  |
| For patients at very high immunological risk, in countries where pre-emptive desensitization for DD recipients is permitted, it is advised to consider pre-emptive desensitization as a means of potentially reducing the risk of AMR, according to local and national guidelines. | 80.60% |
| It is advised to consider pre-emptive desensitization, as a means of further enabling kidney allocation and transplantation among HS patients with the highest unmet need, according to local and national guidelines. | 80.60% |
| **Risk stratification decision tool for imlifidase patient selection** |  |
| ***Primary renal disease*** |  |
| It is advised that primary renal disease not be restrictive when evaluating patients for an HLAi KTx with imlifidase unless it is contraindicated in the imlifidase SmPC. | 80.60% |
| It is advised that patients who are at risk of developing recurrence of original renal disease be assessed individually by the MDT and managed according to local protocols. | 72.20% |
| It is advised that patients who are at risk of developing recurrence of original renal disease be considered as being at very high risk. | 71% |
| It is advised that patients with thrombotic microangiopathy be considered as being at very high risk due to the disease’s early recurrence rate, and thus be approached with extra caution. | 75% |
| It is advised that patients with Primary FSGS (Focal Segmental Glomerulosclerosis) be considered as being at a very high risk due to the disease's high recurrence rate, and thus be approached with extra caution. | 83.30% |
| It is advised that patients with atypical haemolytic uremic syndrome be considered as being at high risk, and thus be approached with caution. | 83% |
| ***Dialysis & transplant history*** |  |
| It is advised that HS patients with a history of severe AMR be considered as being at very high risk of poor outcomes and thus be approached with extra caution. | 84.40% |
| It is advised that HS patients with history of multiple previous kidney transplants be considered as being at high or very high risk, considering the cause of the previous graft loss. | 90.60% |
| It is advised that patients with long dialysis vintage (time on dialysis) be considered as being at high risk due to the increased likelihood of related complications, like atheroma and exacerbated comorbidities, and thus be approached with extra caution. | 66.70% |
| It is advised that patients with exhausted standard routes of vascular access be prioritized for an HLAi KTx due to higher medical urgency. | 80.60% |

Ab: antibody; AMR: antibody-mediated rejection; CDCXM: complement-dependent cytotoxicity crossmatch; DD: deceased donor; DSA: donor-specific antibodies; HLA: human leukocyte antigen; HLAi: HLA incompatible; HS: highly sensitized; KTx: kidney transplant; MDT: multidisciplinary team; MFI: mean fluorescence intensity; SmPC: summary of product characteristics.

**Supplementary Table S4.** Statements and level of agreement regarding donor–recipient profile.

| **Statement** | **% of agreement** |
| --- | --- |
| **Organ quality** | |
| It is advised selecting high quality kidneys from deceased donors that are not considered to be at high risk of failure (e.g., signs of severe acute tubular necrosis), unless there are pressing reasons to consider otherwise. | 77.80% |
| It is advised that the organ quality and function be validated by the recipient transplant center before administrating imlifidase. | 88.90% |
| **Donor-recipient immunological compatibility** | |
| It is advised that the number of HLA mismatches not be an exclusion factor for accepting a donor kidney, provided there is sufficient prior experience in HLAi transplants. | 86.10% |
| Whenever possible, it is advised aiming for fewer mismatches in younger recipients, considering the prospect of potentially needing other transplant(s) in the future. | 86.10% |
| It is advised that, for the same total MFI strength of DSA, patients with increasingly higher numbers of mismatches be considered as being at higher risk. | 72.20% |
| It is advised considering that patients with increasing number of mismatches are at potentially higher risk for long-term rejection. | 86.10% |

DSA: Donor-specific antibodies; HLA: Human leukocyte antigen; HLAi: HLA incompatible; MFI: Mean fluorescence intensity.

**Supplementary Table S5.** Statements and level of agreement regarding imlifidase administration and crossmatch conversion.

| **Statement** | **% of agreement** |
| --- | --- |
| **Donor-specific anti-HLA antibody assessment pre-imlifidase use** | |
| Prior to administering imlifidase, it is advised that donor-recipient immunological compatibility be assessed according to the local lab protocols and capabilities, and to perform:   - Preferably, and if feasible, a SAB assay in addition to a FCXM and a CDCXM, particularly in the early stages of imlifidase experience, to provide more assurance around risk assessment and generate evidence to further support risk stratification and interpretation across patients. - At least a FCXM or a CDCXM paired with a fresh or recent (<6 weeks) SAB assay | 83.90% |
| It is advised to consider the number and type of DSA loci when evaluating risk of a positive physical crossmatch, CDCXM or FCXM. | 88.60% |
| It is advised that each center has pre-defined criteria to determine whether the FCXM is deemed borderline positive, clearly positive, or very positive. It is advised that HLAi KTx with:   - Borderline positive FCXM be transplanted with or without imlifidase, but post-transplant management with higher levels of immunosuppression compared to FCXM negative HLAi KTx - Clearly positive FCXM be considered to be at high immunological risk and be desensitized using imlifidase. - Very positive FCXM (equivalent to positive CDCXM) be considered to be at very high immunological risk and either not proceed with the transplant or be desensitized with imlifidase, provided there are significant pressing reasons and prior experience with HLAi KTx | 77.40% |
| It is advised to consider a positive T-cell CDCXM as very high risk and avoid conducting HLAi KTx following a positive T-cell CDCXM at least in the beginning, while building experience with imlifidase. | 81.30% |
| It is advised that a T-cell FCXM be considered:   - Borderline positive for MCS values of <100 - Clearly positive for MCS values of 100-200 - Very positive for MCS values of 200-250 | 64.50% |
| It is advised that a B-cell FCXM be considered:   - Borderline positive for MCS values of <200 - Clearly positive for MCS values of 200-300 - Very positive for MCS values of 300-350 | 62.10% |
| **Practical considerations of imlifidase administration on cold ischemia time** | |
| It is advised selecting donor kidneys with as short as possible cold ischemia time, preferably of less than 18 hours, unless there are pressing reasons to consider otherwise. | 94.40% |
| **Donor-specific anti-HLA antibody assessment post-imlifidase use** | |
| After using imlifidase, it is advised to assess the crossmatch conversion prior to proceeding with the transplant by doing a physical crossmatch, FCXM or CDCXM, according to local practice. In addition, if possible, it is advised considering performing a Luminex SAB assay post-imlifidase pre-transplantation. | 78.10% |
| Provided there is sufficient time and donor/recipient cells, it is advised to assess the crossmatch conversion via a physical crossmatch, following the second imlifidase dose and prior to proceeding with the transplant. | 82.70% |
| For future research purposes, it would be advised to assess the patient's DSA profile by doing a SAB assay with fresh sera following imlifidase and prior to proceeding with the transplant. | 88.90% |
| It is advised that a second dose of imlifidase be administered within 24 hours after the first dose if the crossmatch has not been converted. | 71% |

CDCXM: Complement-dependent cytotoxicity crossmatch; DSA: Donor-specific antibodies; FCXM: Flow cytometric crossmatch; HLA: Human leukocyte antigen; HLAi: HLA incompatible; KTx: Kidney transplant; SAB: Single-antigen bead.

**Supplementary Table S6.** Statements and level of agreement regarding imlifidase patient post-transplant management.

| **Statement** | **% of agreement** |
| --- | --- |
| **Imlifidase patient monitoring** | |
| It is advised that open communication channels between the local hospital and transplant center be established to ensure best practice protocols are in place around post-transplant management and emergency response. | 87.50% |
| It is advised that patients are kept in the transplant center for as long as possible following the HLAi KTx to ensure close monitoring is conducted and optimal care is provided. | 75% |
| It is advised that monitoring of kidney function, infections and overall clinical status of the patients be conducted according to the KTx standard of care, following local and national guidelines. | 97.10% |
| It is advised that a longer close follow up post-HLAi KTx is arranged, and patients visit the transplant center at regular intervals following their transplant, according to local protocols and their individual risk factor needs, and preferably at least:   - For the first 1-2 months, twice a week - For the following 3-4 months, twice a month - For the following 6 months, once per month for stable patients and twice a month for patients at higher risk of AMR - Once a year, past the first year, provided they regularly visit their local nephrologist | 87.10% |
| It is advised that patients visit their local nephrologist at least once every 3 months during the first year following an HLAi KTx. | 80% |
| For transplanted patients with FSGS, it is advised that proteinuria be closely monitored following the first two weeks of the KTx and if detected, immediately perform a biopsy to determine if the cause is acute ABMR or FSGS recurrence. | 97.20% |
| It is advised to closely monitor the DSA using a SAB assay according to local lab protocols, to increase the likelihood of identifying the DSA rebound as soon as possible to the time it occurs. | 93.80% |
| It is advised to closely monitor the DSA using a SAB assay according to local lab protocols, to increase the likelihood of identifying the Ab rebound as soon as possible to the time it occurs. | 93.80% |
| It would be preferable to assess the DSA following the transplant at:   - Days 3, 5, 7 and 10 (or day 3 and day 7 if DSA testing resourcing is limited) (Not if IVIG given on day 9 & 10) - Months 1, 3 and 6 - Once a year | 87.10% |
| It is advised to adapt the DSA monitoring frequency individually for each patient based on the initial few SAB results. | 71.90% |
| It is advised to consider the potential interference effect of IVIg on SAB assay results and adapt DSA monitoring frequency appropriately. | 81.30% |
| It is advised to consider the potential effect of anti-CD20 treatment on SAB assay results and adapt DSA monitoring frequency appropriately. | 67.70% |
| It is advised that a pre-emptive biopsy is performed prior to hospital discharge according to local protocols to re-evaluate the induction immunosuppressive regimen, preferably. | 51.60% |
| **Anti-microbial strategies** | |
| It is advised that antimicrobial prophylaxis is provided to all patients prior and following HLAi KTx, according to local protocols and their individual risk factor needs. | 96.80% |
| It is advised that antimicrobial prophylaxis is maintained for all patients for at least 4 weeks post-KTx with imlifidase. | 77.40% |
| It is advised that all patients have received vaccination against COVID-19 prior to desensitization, and at least 2 weeks apart from any cell-depleting therapy. | 100% |
| It is advised that all patients be provided COVID-19 monoclonal antibodies as prophylaxis following transplantation. | 35.50% |
| **Immunosuppressive treatment** | |
| It is advised that an immunosuppression induction and maintenance protocol tailored to the needs of HS patients be developed and approved by the core team in advance, according to local clinical practices and access to treatments. | 93.60% |
| It is advised to use steroids for all patients regardless of risk profile and avoid early withdrawal of steroids. | 94.50% |
| It is advised that high doses of immunosuppression, preferably triple-agent regimen (TAC-MMF-CS), is provided to all patients according to local protocols and their individual risk factor needs. | 94.40% |
| It is advised that calcineurin inhibitors are considered as part of the immunosuppression regimen and dose is adjusted according to trough levels as per standard of care protocols. | 100% |
| It is advised that IMDH inhibitors (e.g., MMF) are considered as part of the immunosuppression regime as per standard of care protocols. | 91.70% |
| In cases where rabbit ATG is used over ATGAM, it is advised to start after day 4 after imlifidase use before administrating rATG to patients. | 91.70% |
| In centers where ATG is available and deemed appropriate to use, it is advised that T-cells count be regularly monitored and ATG dose be adjusted accordingly. | 88.60% |
| It is advised that an anti-CD20 is added in the immunosuppression regimen of patients who are at high risk of AMR. | 72.20% |
| It is advised that preferably IVIG is administered to higher risk patients three times a week for 2 weeks following the transplant. | 38.70% |
| It is advised that IL-6 receptor antagonists (if available) are considered as part of the immunosuppression regimen and dose is adjusted according to white blood cells and platelets count as well as liver function status. | 22.20% |
| **AMR management** | |
| It is advised that plasmapheresis is considered as part of the AMR management as per standard of care protocols and the patient’s individual risk factor needs. | 93.80% |
| It is advised that potentially arising immunological complications be managed exclusively by the transplant center regardless of time passed since the HLAi KTx. | 86.10% |
| It is advised that predetermined protocols for the treatment of antibody-mediated, acute, and chronic, rejection be well defined in advance and ready in place if planning to use imlifidase, according to national and local guidelines, to ensure immediate clinical response. | 91.70% |
| It is advised that predetermined protocols for the treatment of T-cell mediated rejection, acute and chronic, be well defined in advance and ready in place if planning to use imlifidase, according to national and local guidelines, to ensure immediate clinical response. | 94.50% |
| It is advised that, in case of acute unprecedentedly high antibody production during the first week post-transplantation with normal creatinine levels, urgent AMR intervention is carried out, a biopsy be performed, and results made available in a timely manner for evaluation and treatment. | 86.10% |
| It is advised to perform biopsies in time-critical circumstances and cases of severely impaired renal function and AMR suspicion, to directly proceed with anti-rejection treatment, prior to performing or receiving results from a biopsy. | 96.80% |
| It is advised that the management of AMR should be similar to a standard kidney transplant, following local AMR protocols, but implemented earlier with a more rapid stepwise approach, incorporating complement inhibitor and/or anti-IL6 earlier, if needed. If AMR is not appropriately managed, consider alternative options such as splenectomy. | 87.10% |

ABMR: Antibody-mediated acute rejection; Ab: Antibody; AMR: Antibody-mediated rejection; ATG: Antithymocyte globulin; ATGAM: Equine anti-thymocyte globulin; DSA: Donor-specific antibodies; FSGS: Focal segmental glomerulosclerosis; HLAi: HLA incompatible; IL-6: Interleukin 6; IMDH: Inosine monophosphate dehydrogenase; IVIg: Intravenous immunoglobulin; KTx: Kidney transplant; MMF: Mycophenolate mofetil; rATG: Rabbit anti-human thymocyte globulin; SAB: Single-antigen bead; TAC-MMF-CS: TAC Tacrolimus - MMF mycophenolate mofetil - CS  Corticosteroid.
